# How low can you go? Intraoperative microdosing of indocyanine green for fluorescence cholangiography during laparoscopic cholecystectomy

**DOI:** 10.1007/s00464-025-12057-w

**Published:** 2025-08-11

**Authors:** Ryan C. Broderick, Graham J. Spurzem, Ana Garcia Cabrera, Patricia Ruiz-Cota, Amanda Rocha, Edgardo Reyes, Agustina Altolaguirre, Andres Fontaine-Nicola, Hannah M. Hollandsworth, Bryan J. Sandler, Santiago Horgan, Garth R. Jacobsen

**Affiliations:** Department of Surgery, Division of Minimally Invasive Surgery, University of California San Diego, 9300 Campus Point Dr., La Jolla, San Diego, CA 92037 USA

**Keywords:** Laparoscopic cholecystectomy, Indocyanine green, Fluorescence cholangiography, Minimally invasive surgery, Microdose

## Abstract

**Background:**

Fluorescence cholangiography (FC) with indocyanine green (ICG) enables real-time intraoperative visualization of extrahepatic biliary anatomy during laparoscopic cholecystectomy (LC). There is no consensus on the optimal ICG dose or timing of administration for LC. The goal of this study was to implement a simple intraoperative low-dose (“microdose”) ICG protocol that facilitates non-inferior biliary visualization compared to a standard dose protocol to streamline perioperative workflows.

**Methods:**

A retrospective review of a prospectively maintained database identified patients who underwent LC at our institution from 2021 to 2024. Microdose protocol patients were intravenously administered 0.5 mg ICG upon induction of general anesthesia, while standard protocol patients received 7.5 mg ICG 60–120 min preoperatively. Operative video from cases in both groups were reviewed to compare the frequency of cystic duct (CD), common bile duct (CBD), and common hepatic duct (CHD) visualization with FC. A 4-point Likert scale survey was completed for each case to compare ICG signal strength (1 = no signal; 4 = very strong), clarity from background interference (1 = cannot discern signal from background; 4 = no interference), and usefulness in clinical decision-making (1 = no benefit; 4 = essential).

**Results:**

A total of 100 patients were identified (*N* = 50 microdose; *N* = 50 standard). For microdose cases, the mean time from ICG administration to skin incision was 16.7 ± 5.6 min, and mean operative time was 47.4 ± 20.2 min. Compared to 50 standard dose cases, CD visualization before hepatocystic triangle dissection with the microdose protocol was 86.0% vs 88.0% (*p* = 0.99). The CBD and CHD were seen in all cases for both groups. There were no significant differences in average score for signal strength (3.6 ± 0.5 vs 3.7 ± 0.5, *p* = 0.32), signal clarity (3.4 ± 0.5 vs 3.3 ± 0.5, *p* = 0.32), or usefulness in clinical decision-making (4.0 ± 0.0 vs 4.0 ± 0.0, *p* = 0.99) between groups.

**Conclusion:**

Microdose ICG can be given intraoperatively to provide excellent biliary visualization during laparoscopic cholecystectomy, eliminating the historical workflow of preoperative ICG administration.

Laparoscopic cholecystectomy (LC) is one of the most commonly performed surgical procedures in the United States, with approximately 750,000 performed annually [1]. Despite widespread adoption of LC for benign gallbladder pathology, devastating intraoperative complications such as common bile duct injury (CBDI) continue to occur. The reported incidence of CBDI is generally recognized to be higher in LC compared to open cholecystectomy, with rates ranging from 0.03 to 2.6% across studies [2–4]. The primary cause of CBDI is misinterpretation of biliary anatomy in 71 to 97% of cases [5, 6]. Intraoperative cholangiogram (IOC) has been recommended to aid in anatomy identification, but its routine use is debated [1, 7, 8]. Fluorescence cholangiography (FC) with near-infrared (NIR) imaging after intravenous injection of indocyanine green (ICG) dye is an alternative technique that enables real-time intraoperative identification of extrahepatic biliary anatomy while avoiding ionizing radiation.

Routine use of ICG during LC has been shown to decrease operative time, conversion to open rates, and shorten hospital length of stay compared to standard white light laparoscopy [9, 10]. Fluorescence cholangiography with ICG has also been confirmed as an efficient and cost-effective surgical strategy [11]. However, there is no consensus on the optimal ICG dose or timing of administration for LC. Most studies use 2.5 mg of ICG administered approximately 1 h before surgery, though protocols vary widely in the literature [12]. Recent case series and prospective studies have investigated lower ICG dose protocols to decrease interference from background liver fluorescence and improve biliary visualization [13–15]. NIR camera sensitivity and intraoperative displays have also significantly improved since the inception of FC, potentially decreasing the ICG dose and timing of administration required for adequate biliary visualization.

Our institution has historically administered 7.5 mg of ICG approximately 1 to 2 h preoperatively, a protocol developed with older generation camera systems. Since the implementation of our protocol, more advanced 4K camera platforms have been released with greater image sensor sensitivity. The goal of this study was to implement a simple intraoperative low-dose (“microdose”) ICG protocol in conjunction with these newer camera systems to achieve non-inferior biliary visualization compared to our standard protocol. We hypothesize that administering low-dose ICG at the time of general anesthesia induction will enable comparable biliary visualization, thereby simplifying our perioperative workflows and increasing flexibility in surgical planning.

## Methods

### Study design

A retrospective review of a prospectively maintained database identified patients who underwent LC at our institution from 2021 to 2024. Patients in the microdose group were intravenously administered 0.5 mg of ICG upon induction of general anesthesia, while patients in the standard group received 7.5 mg of ICG approximately 60 to 120 min preoperatively. Operative videos from cases in both groups were retrospectively reviewed by the operating surgeons to compare the frequency of cystic duct (CD), common bile duct (CBD), and common hepatic duct (CHD) visualization with FC. A 4-point Likert scale survey was completed for each case to compare ICG signal strength (1 = no signal; 4 = very strong), clarity from background interference (1 = cannot discern signal from background; 4 = no interference), and usefulness in clinical decision-making (1 = no benefit; 4 = essential).

Patient demographics and perioperative outcomes between the microdose and standard groups were compared. Demographics included age, sex, body mass index (BMI), American Society of Anesthesiologists (ASA) classification, Charlson Comorbidity Index (CCI), preoperative diagnosis, and final pathology results. Operative variables included time from ICG administration to skin incision and operative difficulty. The Nassar scale was used to grade operative difficulty for each case based on operative findings of the gallbladder, cystic pedicle, and associated adhesions [16]. The grading system is designed to be used as an overall summary of the operative conditions found, with the worst score among the 3 categories serving as the overall grade for each individual case. Outcomes included operative time, conversion to open rate, intraoperative complications, hospital length of stay (LOS), and 30-day morbidity, mortality, readmission, and reintervention rates. Any aberrant biliary anatomy identified intraoperatively was also noted.

### ICG protocols and equipment

For all cases, one 25 mg vial of ICG was reconstituted in 10 mL of sterile water. After reconstitution, a 25 mg vial of ICG contains 2.5 mg of dye per 1 mL of solution. For the microdose protocol, 0.2 mL of this solution (0.5 mg ICG) was administered intravenously upon induction of general anesthesia in the operating room. For the standard protocol, 3 mL of this solution (7.5 mg ICG) was administered intravenously approximately 60 to 120 min before surgery in the preoperative area. Microdose cases were performed using a 4K laparoscopic camera platform (SynergyID™, Arthrex, Inc, Naples, FL) equipped with NIR fluorescence imaging capabilities.

### Statistical analysis

Categorical variables were reported as a frequency and percentage. Continuous variables were reported as a mean and standard deviation (SD). Patient demographics, operative variables, perioperative outcomes, biliary visualization, and results of the Likert scale survey were compared between the microdose and standard groups. Pearson chi-squared test or Fisher’s exact test were used to compare categorical variables as appropriate. Independent sample t-testing was used for comparison of continuous variables. A *p* value of < 0.05 was considered statistically significant. All statistical analyses were performed using R (Version 4.4.1; Vienna, Austria).

## Results

### Patient demographics and operative variables

A total of 100 patients were included in the analysis (*N* = 50 microdose; *N* = 50 standard dose). There were no significant differences between groups in terms of age, sex, BMI, ASA classification, CCI, preoperative diagnosis, final pathology results, and Nassar score (Table 1). The majority of procedures were performed for a preoperative diagnosis of symptomatic cholelithiasis in both groups. The mean time from ICG administration to skin incision was significantly shorter in the microdose group compared to the standard dose group (16.7 ± 5.6 vs 94.9 ± 37.6 min, *p* < 0.001).

**Table 1:** Patient demographics and operative variables

Patient feature	Microdose (*N* = 50)	Standard dose (*N* = 50)	*p* value
Age (years), mean ± SD	49.7 ± 16.6	50.2 ± 16.7	0.88
Sex, *N* (%)			0.32
Female	42 (84.0)	38 (76.0)	
Male	8 (16.0)	12 (24.0)	
BMI (kg/m^2^), mean ± SD	27.3 ± 4.5	29.2 ± 6.8	0.10
ASA class, *N* (%)			0.46
1	3 (6.0)	1 (2.0)	
2	32 (64.0)	30 (60.0)	
3	15 (30.0)	19 (38.0)	
CCI, mean ± SD	1.6 ± 1.6	1.6 ± 1.5	0.99
Preoperative diagnosis, *N* (%)			0.80
Cholelithiasis	37 (74.0)	33 (66.0)	
Acute cholecystitis	1 (2.0)	3 (6.0)	
Chronic cholecystitis	5 (10.0)	4 (8.0)	
Gallbladder polyp	2 (4.0)	5 (10.0)	
Gallstone pancreatitis	1 (2.0)	1 (2.0)	
Choledocholithiasis	2 (4.0)	3 (6.0)	
Biliary dyskinesia	1 (2.0)	1 (2.0)	
Cholangitis	1 (2.0)	0 (0.0)	
Pathology results, *N* (%)			0.38
Acute on chronic cholecystitis	4 (8.0)	4 (8.0)	
Chronic cholecystitis	40 (80.0)	40 (80.0)	
Cholelithiasis	4 (8.0)	1 (2.0)	
Cholesterol polyp	2 (4.0)	5 (10.0)	
Time from ICG to incision (min), mean ± SD	16.7 ± 5.6	94.9 ± 37.6	** < 0.001**
Nassar score, mean ± SD	2.1 ± 0.5	2.0 ± 0.6	0.37

*p* values in bold indicate statistical significance

*SD* standard deviation, *BMI* body mass index, *ASA* American Society of Anesthesiologists, *CCI* Charlson Comorbidity Index, *ICG* indocyanine green

### Biliary visualization

Compared to 50 standard dose cases, the frequency of CD visualization before dissection of the hepatocystic triangle with the microdose protocol was 86.0% vs 88.0% (*p* = 0.99). The CBD and CHD were visualized in all cases for both groups. Results of the Likert scale survey revealed no significant differences in average score for signal strength (3.6 ± 0.5 vs 3.7 ± 0.5, *p* = 0.32), signal clarity (3.4 ± 0.5 vs 3.3 ± 0.5, *p* = 0.32), or usefulness in clinical decision-making (4.0 ± 0.0 vs 4.0 ± 0.0, *p* = 0.99) between groups (Table 2).

**Table 2:** Biliary visualization

Variable	Microdose (*N* = 50)	Standard dose (*N* = 50)	*p* value
Structure visualized, *N* (%)			0.99
Cystic duct	43 (86.0)	44 (88.0)	
Common hepatic duct	50 (100.0)	50 (100.0)	
Common bile duct	50 (100.0)	50 (100.0)	
Likert scale, mean ± SD			
ICG signal strength	3.6 ± 0.5	3.7 ± 0.5	0.32
Clarity from background interference	3.4 ± 0.5	3.3 ± 0.5	0.32
Usefulness in clinical decision-making	4.0 ± 0.0	4.0 ± 0.0	0.99

*SD* standard deviation, *ICG* indocyanine green

### Perioperative outcomes

There were no significant differences between groups in terms of 30-day outcomes, including morbidity, mortality, readmission, and reintervention rates (Table 3). Two patients in the microdose group developed postoperative superficial incisional surgical site infections that were treated with oral antibiotics. Another patient in the microdose group developed a postoperative acute kidney injury requiring readmission for fluid resuscitation. Two patients in the standard dose group developed postoperative intra-abdominal abscesses requiring readmission and drainage by interventional radiology. Operative time and hospital LOS were also similar between groups. We identified four cases (8.0%) of aberrant biliary anatomy in the microdose group. There were no conversions to open or intraoperative complications in either group.

**Table 3:** Perioperative outcomes

Outcome	Microdose (*N* = 50)	Standard dose (*N* = 50)	*p* value
30-day outcomes, *N* (%)			0.99
Morbidity	3 (6.0)	2 (4.0)	
Mortality	0 (0.0)	0 (0.0)	
Readmission	1 (2.0)	2 (4.0)	
Reintervention	0 (0.0)	2 (4.0)	
Operative time, mean ± SD	47.4 ± 20.2	52.1 ± 18.3	0.23
Conversion to open, *N* (%)	0 (0.0)	0 (0.0)	–
Intraoperative complications, *N* (%)	0 (0.0)	0 (0.0)	–
Hospital length of stay, mean ± SD	0.2 ± 0.4	0.1 ± 0.3	0.16
Aberrant biliary anatomy, *N* (%)	4 (8.0)	0 (0.0)	0.12

*SD* standard deviation

## Discussion

Despite the widespread use of LC for benign gallbladder pathology, ICG cholangiography has not yet become standard of care as an adjunct to the critical view of safety. Our institution has previously shown the benefits of ICG for LC and adopted this strategy for routine use. [9, 10] There is currently no consensus on the ICG dose or timing of administration that facilitates optimal intraoperative biliary visualization. In this study, we demonstrate that implementation of a low-dose ("microdose") ICG protocol provides non-inferior biliary visualization compared to our historical standard protocol with equivalent operative time and perioperative outcomes. Simplifying the ICG protocol for LC may help to streamline perioperative workflows and increase adoption of the technique.

Reported ICG protocols vary widely in the literature. A randomized trail published in 2020 by Chen et al. asserted that the optimal effect of FC is achieved by performing 10 mg ICG injections 10 to 12 h prior to surgery [17]. Conversely, more recent prospective studies have investigated lower ICG dose protocols. Ladd et al. randomized 55 adult patients to receive either 0.05 mg or 0.25 mg of ICG on induction of anesthesia and found that the lower dose led to a quantitative improvement in biliary visualization by minimizing background liver fluorescence [14]. The study also found the low-dose protocol provided satisfactory qualitative visualization of major extrahepatic biliary anatomy. Another recent randomized trial using a 4 K fluorescent imaging system for FC found that ICG doses ranging from 10 to 25 µg (0.01 to 0.025 mg) given within 30 min preoperatively provided adequate biliary visualization [18]. Other groups have advocated for weight-based ICG dosing and even transhepatic intracholecystic injection of ICG in select patients [19, 20]. While ICG cholangiography is efficacious across a range of strategies, intraoperative administration of low-dose ICG likely provides the most streamlined and cost-effective approach. ICG administration several hours prior to surgery is often not feasible for planned outpatient cholecystectomy. In addition, broad use of lower ICG doses may help to alleviate potential ICG shortages by reducing waste, and intraoperative administration removes an additional step in nursing workflows [21]. The demonstrated efficacy of low-dose ICG at the time of induction also enables redosing intraoperatively for unsatisfactory visualization as needed.

Although the CHD and CBD were identified in all cases for both groups, there were several instances in which we observed sluggish CD filling or no CD filling altogether. One patient in the microdose group who had previously received hepatotoxic chemotherapy for a hematologic malignancy had no CD filling at 18 min after ICG administration; however, we observed complete CD filling in real time over the next several minutes without having to give additional ICG. There were also several patients in both groups with cystic duct obstruction secondary to chronic cholecystitis who did not achieve CD filling during surgery. Pharmacokinetic studies have shown a measurable decrease in ICG hepatic clearance in patients with cirrhosis due to a decrease in intrinsic liver function [22]. ICG intrinsic hepatic clearance is also an independent predictor of survival in cirrhosis [23]. Biliary excretion of ICG is subsequently delayed due to decreased hepatic clearance, leading some studies to recommend intracholecystic ICG injection for patients with cirrhosis and fatty liver disease [20, 24]. In our experience, waiting a number of minutes for complete biliary excretion of ICG was sufficient for visualization in patients with decreased liver function. Therefore, there may be a role for modestly delaying ICG administration in patients with suspected liver impairment to account for reduced hepatic clearance and prevent prolonged operative time. In addition, despite not being able to visualize the CD in some patients with chronic gallbladder inflammation, the CBD and CHD were always visible and facilitated our ability to safely complete each case using these structures as key landmarks.

The rapid growth of fluorescence-guided surgery (FGS) as a field is demonstrated by the release of several flagship fluorescence imaging devices by large medical device companies. A recent systematic review identified 10 devices developed specifically for minimally invasive surgery, each designed to work within the ICG wavelength of 780 to 830 nm [25]. Each system provides 3 to 4 similar imaging modes, such as monochromatic, green fluorescence overlay, fluorescence only, and gradient overlay depending on signal intensity. These devices represent a significant advancement from early investigations into laparoscopic infrared imaging dating back to the inception of laparoscopic surgery [26]. As camera sensor and imaging technology continue to advance, clinical protocols for the use of fluorescent agents will likely need to adapt. There may also be a role for manufacturers of these fluorescent agents to modify the production of injection kits, as propagation of more sensitive imaging systems will likely result in less dye being needed in each kit to achieve the same degree of visualization. Alternatively, efforts could also be made to allow one vial of a fluorescent dye to be used for multiple patients to reduce waste and cost.

There are several limitations to this study, namely its retrospective single-center design and lack of randomization. Older generation camera systems were used for standard dose cases, which could feasibly impact the qualitative assessment performed. Moreover, video reviewers could not be blinded to the ICG dose given, as there was a clear identifiable difference in operative video quality between systems. Further study is necessary to examine the effect of various patient factors on biliary visualization using this protocol, such as inflammation, body mass index, and visceral fat. Validation of low-dose ICG protocols in a larger sample of various patient presentations will also be necessary to support adoption.

## Conclusion

Microdose ICG can be given intraoperatively to provide excellent biliary visualization during laparoscopic cholecystectomy, eliminating the historical workflow of preoperative ICG administration and increasing flexibility in surgical planning.

## References

[1] Flum DR, Dellinger EP, Cheadle A, Chan L, Koepsell T (2003) Intraoperative cholangiography and risk of common bile duct injury during cholecystectomy. JAMA 289(13):1639–1644 12672731 10.1001/jama.289.13.1639

[2] Kohn JF, Trenk A, Kuchta K, Lapin B, Denham W, Linn JG, Haggerty S, Joehl R, Ujiki MB (2018) Characterization of common bile duct injury after laparoscopic cholecystectomy in a high-volume hospital system. Surg Endosc 32(3):1184–1191 28840410 10.1007/s00464-017-5790-8

[3] Stefanidis D, Chintalapudi N, Anderson-Montoya B, Oommen B, Tobben D, Pimentel M (2017) How often do surgeons obtain the critical view of safety during laparoscopic cholecystectomy? Surg Endosc 31(1):142–146 27142437 10.1007/s00464-016-4943-5

[4] Aziz H, Pandit V, Joseph B, Jie T, Ong E (2015) Age and obesity are independent predictors of bile duct injuries in patients undergoing laparoscopic cholecystectomy. World J Surg 39(7):1804–1808 25663013 10.1007/s00268-015-3010-z

[5] Way LW, Stewart L, Gantert W, Liu K, Lee CM, Whang K, Hunter JG (2003) Causes and prevention of laparoscopic bile duct injuries: analysis of 252 cases from a human factors and cognitive psychology perspective. Ann Surg 237(4):460–469 12677139 10.1097/01.SLA.0000060680.92690.E9 PMC1514483

[6] Pesce A, Portale TR, Minutolo V, Scilletta R, Li Destri G, Puleo S (2012) Bile duct injury during laparoscopic cholecystectomy without intraoperative cholangiography: a retrospective study on 1,100 selected patients. Dig Surg 29(4):310–314 22986956 10.1159/000341660

[7] Ishizawa T, Bandai Y, Ijichi M, Kaneko J, Hasegawa K, Kokudo N (2010) Fluorescent cholangiography illuminating the biliary tree during laparoscopic cholecystectomy. Br J Surg 97(9):1369–1377 20623766 10.1002/bjs.7125

[8] Dip FD, Asbun D, Rosales-Velderrain A, Lo Menzo E, Simpfendorfer CH, Szomstein S, Rosenthal RJ (2014) Cost analysis and effectiveness comparing the routine use of intraoperative fluorescent cholangiography with fluoroscopic cholangiogram in patients undergoing laparoscopic cholecystectomy. Surg Endosc 28(6):1838–1843 24414461 10.1007/s00464-013-3394-5

[9] Broderick RC, Lee AM, Cheverie JN, Zhao B, Blitzer RR, Patel RJ, Soltero S, Sandler BJ, Jacobsen GR, Doucet JJ, Horgan S (2021) Fluorescent cholangiography significantly improves patient outcomes for laparoscopic cholecystectomy. Surg Endosc 35(10):5729–5739 33052527 10.1007/s00464-020-08045-x

[10] Broderick RC, Li JZ, Huang EY, Blitzer RR, Lee AM, Serra JL, Bouvet M, Sandler BJ, Jacobsen GR, Horgan S (2022) Lighting the way with fluorescent cholangiography in laparoscopic cholecystectomy: reviewing 7 years of experience. J Am Coll Surg 235(5):713–723 36102574 10.1097/XCS.0000000000000314

[11] Reeves JJ, Broderick RC, Lee AM, Blitzer RR, Waterman RS, Cheverie JN, Jacobsen GR, Sandler BJ, Bouvet M, Doucet J, Murphy JD, Horgan S (2022) The price is right: routine fluorescent cholangiography during laparoscopic cholecystectomy. Surgery 171(5):1168–1176 34952715 10.1016/j.surg.2021.09.027

[12] Serban D, Badiu DC, Davitoiu D, Tanasescu C, Tudosie MS, Sabau AD, Dascalu AM, Tudor C, Balasescu SA, Socea B, Costea DO, Zgura A, Costea AC, Tribus LC, Smarandache CG (2022) Systematic review of the role of indocyanine green near-infrared fluorescence in safe laparoscopic cholecystectomy (review). Exp Ther Med 23(2):187 35069868 10.3892/etm.2021.11110 PMC8764893

[13] Pujol-Cano N, Molina-Romero FX, Palma-Zamora E, Bonnin-Pascual J, Coll-Sastre M, González-Argenté FX, Morón-Canis JM (2022) Near-infrared fluorescence cholangiography at a very low dose of indocyanine green: quantification of fluorescence intensity using a colour analysis software based on the RGB color model. Langenbecks Arch Surg 407(8):3513–3524 35879621 10.1007/s00423-022-02614-5

[14] Ladd AD, Zarate Rodriguez J, Lewis D, Warren C, Duarte S, Loftus TJ, Nassour I, Soma D, Hughes SJ, Hammill CW, Zarrinpar A (2023) Low vs standard-dose indocyanine green in the identification of biliary anatomy using near-infrared fluorescence imaging: a multicenter randomized controlled trial. J Am Coll Surg 236(4):711–717 36728303 10.1097/XCS.0000000000000553

[15] Zarate Rodriguez JG, Hammill CW (2021) Micro-dosing of indocyanine green for intraoperative fluorescence cholangiography. Surg Technol Int 38:98–101 33724433

[16] Griffiths EA, Hodson J, Vohra RS, Marriott P, Katbeh T, Zino S, Nassar AHM (2019) Utilisation of an operative difficulty grading scale for laparoscopic cholecystectomy. Surg Endosc 33(1):110–121 29956029 10.1007/s00464-018-6281-2 PMC6336748

[17] Chen Q, Zhou R, Weng J, Lai Y, Liu H, Kuang J, Zhang S, Wu Z, Wang W, Gu W (2021) Extrahepatic biliary tract visualization using near-infrared fluorescence imaging with indocyanine green: optimization of dose and dosing time. Surg Endosc 35(10):5573–5582 33026517 10.1007/s00464-020-08058-6 PMC8437885

[18] Liu H, Kuang J, Xu Y, Li T, Li P, Huang Z, Zhang S, Weng J, Lai Y, Wu Z, Lin F, Gu W, Huang Y (2023) Investigation of the optimal indocyanine green dose in real-time fluorescent cholangiography during laparoscopic cholecystectomy with an ultra-high-definition 4K fluorescent system: a randomized controlled trial. Update Surg 75(7):1903–1910 10.1007/s13304-023-01557-w PMC10543949 37314620

[19] Baldari L, Boni L, Kurihara H, Cassinotti E (2023) Identification of the ideal weight-based indocyanine green dose for fluorescent cholangiography. Surg Endosc 37(10):7616–7624 37474826 10.1007/s00464-023-10280-x

[20] Elmeligy HA, Hassan HF, Amer MS, Ossama Y, Maher MA, Azzam AM, Rady M (2024) Intravenous injection versus transhepatic intracholecystic injection of indocyanine green (ICG) to outline biliary tree during laparoscopic cholecystectomy. BMC Surg 24(1):330 39455983 10.1186/s12893-024-02612-y PMC11515391

[21] Society of Gynecologic Oncology (2024) SGO clinical practice advisory: indocyanine green (ICG) dye national shortage. https://www.sgo.org/news/indocyanine-green-icg-dye/. Accessed 21 Jan 2025

[22] Kawasaki S, Sugiyama Y, Iga T, Hanano M, Sanjo K, Beppu T, Idezuki Y (1985) Pharmacokinetic study on the hepatic uptake of indocyanine green in cirrhotic patients. Am J Gastroenterol 80(10):801–806 4036939

[23] Merkel C, Bolognesi M, Finucci GF, Angeli P, Caregaro L, Rondana M, Gatta A (1989) Indocyanine green intrinsic hepatic clearance as a prognostic index of survival in patients with cirrhosis. J Hepatol 9(1):16–22 2768794 10.1016/0168-8278(89)90070-6

[24] Cai Y, Chen Q, Cheng K, Chen Z, Wu S, Wu Z, Wang X, Li Y, Balla A, Singh A, Cai H, Gao P, Cai Y, Peng B (2024) Intragallbladder versus intravenous indocyanine green (ICG) injection for enhanced bile duct visualization by fluorescent cholangiography during laparoscopic cholecystectomy: a retrospective cohort study. Gland Surg 13(9):1628–1638 39421052 10.21037/gs-24-198 PMC11480876

[25] Preziosi A, Cirelli C, Waterhouse D, Privitera L, De Coppi P, Giuliani S (2024) State of the art medical devices for fluorescence-guided surgery (FGS): technical review and future developments. Surg Endosc 38(11):6227–6236 39294317 10.1007/s00464-024-11236-5 PMC11525393

[26] Roberts WW, Dinkel TA, Schulam PG, Bonnell L, Kavoussi LR (1997) Laparoscopic infrared imaging. Surg Endosc 11(12):1221–1223 9373300 10.1007/s004649900575

